# Gα_s_, adenylyl cyclase, and their relationship to the diagnosis and treatment of depression

**DOI:** 10.3389/fphar.2022.1012778

**Published:** 2022-11-18

**Authors:** Jeffrey M. Schappi, Mark M. Rasenick

**Affiliations:** ^1^ Departments of Physiology and Biophysics, University of Illinois at Chicago, Chicago, IL, United States; ^2^ Jesse Brown VAMC, Chicago, IL, United States; ^3^ Department of Psychiatry, University of Illinois at Chicago, Chicago, IL, United States; ^4^ Pax Neuroscience, Glenview, IL, United States

**Keywords:** GPCR, cAMP, lipid rafts, cytoskeleton, depression, antidepressant

## Abstract

The relationship between depression, its etiology and therapy, and the cAMP signaling system have been studies for decades. This review will focus on cAMP, G proteins and adenylyl cyclase and depression or antidepressant action. Both human and animal studies are compared and contrasted. It is concluded that there is some synteny in the findings that cAMP signaling is attenuated in depression and that this is reversed by successful antidepressant therapy. The G protein that activates adenylyl cyclase, Gα_s_, appears to have diminished access to adenylyl cyclase in depression, and this is rectified by successful antidepressant treatment. Unfortunately, attempts to link specific isoforms of adenylyl cyclase to depression or antidepressant action suffer from discontinuity between human and animal studies.

## Introduction

The World Health Organization states that major depressive disorder (MDD) is the most common cause of disability worldwide. The medical and non-medical costs of MDD in the US are now estimated at nearly $300B. The current COVID-19 pandemic is likely to exacerbate this. Treatment of MDD also poses significant obstacles. Despite undergoing multiple treatment regimes, about one-third of patients never achieve remission, and it is this “treatment-resistant” group who are at the greatest risk of suicide. Thus, there remains a pressing need for novel compounds that are effective in this nonresponsive population. New antidepressant drugs are needed, and in order to develop them, greater insight is needed into the biology of depression and antidepressant response. Additionally, diagnosis of depression is both difficult, imprecise, and based on subjective inventories. Both diffusely-targeted therapy and imprecise diagnosis are a result of our failure to understand the molecular and cellular biology of depression. Discovery and verification of cellular hallmarks (biomarkers) for both depression and antidepressant response is a pressing need.

### cAMP, BDNF, and depression

No common mechanism has emerged to link the activities of the diverse compounds used in therapy for depression. Although not necessarily linked to therapeutic action, most antidepressants elevate cAMP production and evoke a cascade of events resulting from sustained increase in cAMP (e.g. increased P-CREB and BDNF) (Donati and Rasenick, 2003). Antidepressant treatment also causes a shift in the localization of the heterotrimeric G protein, Gα_s_, from lipid rafts to more “fluid” membrane regions, facilitating Gα_s_ activation of adenylyl cyclase (AC). Both diminished Gα_s_-adenylyl cyclase coupling and an increase in the proportion of Gα_s_ in lipid rafts are seen in depression (post-mortem and peripheral tissue) (Donati et al., 2008; Singh et al., 2020; Targum et al., 2022), and this is consistent with the augmentation of cAMP production by antidepressants. There are also compounds, such as ketamine, and, perhaps psychedelics, that appear to exert more rapid effects on depression, acting in hours rather than weeks for traditional antidepressants. Ketamine shows similar, but more rapid, effects on Gα_s_ and cAMP compared to traditional antidepressants (Wray et al., 2018).

Several studies (*vide infra*) indicate that chronic antidepressant treatment increases physical coupling between Gα_s_ and adenylyl cyclase, resulting in increased cAMP generation. This is consistent with the observation that chronic treatment with antidepressants results in long-term increases in cellular cAMP (Malberg and Blendy, 2005). Consistent with this, depressed subjects show decreased ^11^C rolipram binding that recovers with successful antidepressant treatment (Fujita et al., 2016). Furthermore, increasing cAMP with inhibitors of phosphodiesterase have showed a promising antidepressant-adjuvant properties in a recent clinical study (Zhao et al., 2003; El-Haggar et al., 2018).

The initial studies showing that CREB knockout blocks the behavioral response to antidepressants date back at least 20 years (Conti et al., 2002) and more recent papers target serotonergic and noradrenergic neurons in achieving this effect (Rafa–Zabłocka et al., 2017). BDNF and TrkB knockout also ablated antidepressant effects in mice (Björkholm and Monteggia, 2016). Both humans and mice with the BDNF val66met allele are more vulnerable to stress-induced anxiety and depression, but this is variable with age and sex (Hwang et al., 2006) (Verhagen et al., 2010). A polymorphism in the regulatory region of the human *BDNF* gene, which reduces BDNF expression and release, is also associated with depression (Björkholm and Monteggia, 2016).

Activated by phosphorylation, pCREB in combination with coactivator CPB (CREB binding protein) is able to act as a transcription factor at CRE (cAMP response element), promoting transcription of cAMP-regulated genes, particularly BDNF (brain-derived neurotrophic factor) (Blendy, 2006; Dwivedi and Pandey, 2008). Animal models of stress and depression-like behavior have revealed decreased BDNF expression, as well as loss of synaptic plasticity, particularly in the hippocampus (Duman et al., 1999), as well as restoration of BDNF expression with extended antidepressant treatment (Fujimaki et al., 2000; Coppell et al., 2003; Foubert et al., 2004). Likewise, human postmortem samples show decreases in BDNF expression in depression (Dunham et al., 2009; Castrén and Monteggia, 2021), and increases with antidepressant treatment (Chen et al., 2001). BDNF itself may be required for the action of antidepressants (Adachi et al., 2008), including ketamine (Autry et al., 2011). The decreased expression of BDNF in stress models and in depression, and restoration of BDNF expression with antidepressant treatment, could be linked by the cAMP changes in depression, and with antidepressant treatment, noted above.

## Antidepressants and cAMP

### Monoamine centric antidepressants

The majority of extant antidepressants are targeted at monoamine reuptake and metabolism; particularly, inhibition of these. More recently, drugs targeting melatonergic (agomelatine) (Kennedy and Eisfeld, 2007) and glutamatergic (rapid acting antidepressant ketamine) (Matveychuk et al., 2020) systems have been developed or approved for use in depression. Nonetheless, reuptake inhibitors acting at various combinations of SERT, NET, and DAT (serotonin, norepinephrine, and dopamine reuptake transporters) remain the predominant antidepressant class in clinical use. Reuptake inhibitors have shown, collectively, relevant affinities at numerous sites, including reuptake transporters, their canonical targets, as well as monoamine and cholinergic receptors. Despite 60 + years of research, no clear direct mechanism of action has emerged to link their activities. In the case of serotonin, perhaps the most widely implicated neurotransmitter in depression in lay and scientific press alike, a recent review found no clear association between serotonin and depression and also cited several studies demonstrating decreased serotonin content in human and animal subjects post-antidepressant treatment (Moncrieff et al., 2022).

Sulser and his colleagues suggested that one role of extended treatment with tricyclic antidepressants was desensitization of the β−adrenergic receptor and a generalized dampening of cAMP signaling (Sulser et al., 1984). This was contradicted, in part, by results of Menkes et al., showing augmented cAMP signaling in multiple rat brain regions (but not other tissues) after 3 weeks of antidepressant treatment (including ECS) in rats (Menkes et al., 1983). The apparent controversy was resolved through the use of a cellular model system for antidepressant activity, where the time required for an antidepressant response was 3 days (vs 3 weeks in rats and 8 weeks in humans). In this system, antidepressant exposure desensitized the β-receptor on a much faster timescale (24 h) that required for augmented Gα_s_-activation of AC (Chen and Rasenick, 1995b).

### Model systems for determining antidepressant action

**FIGURE 1:**
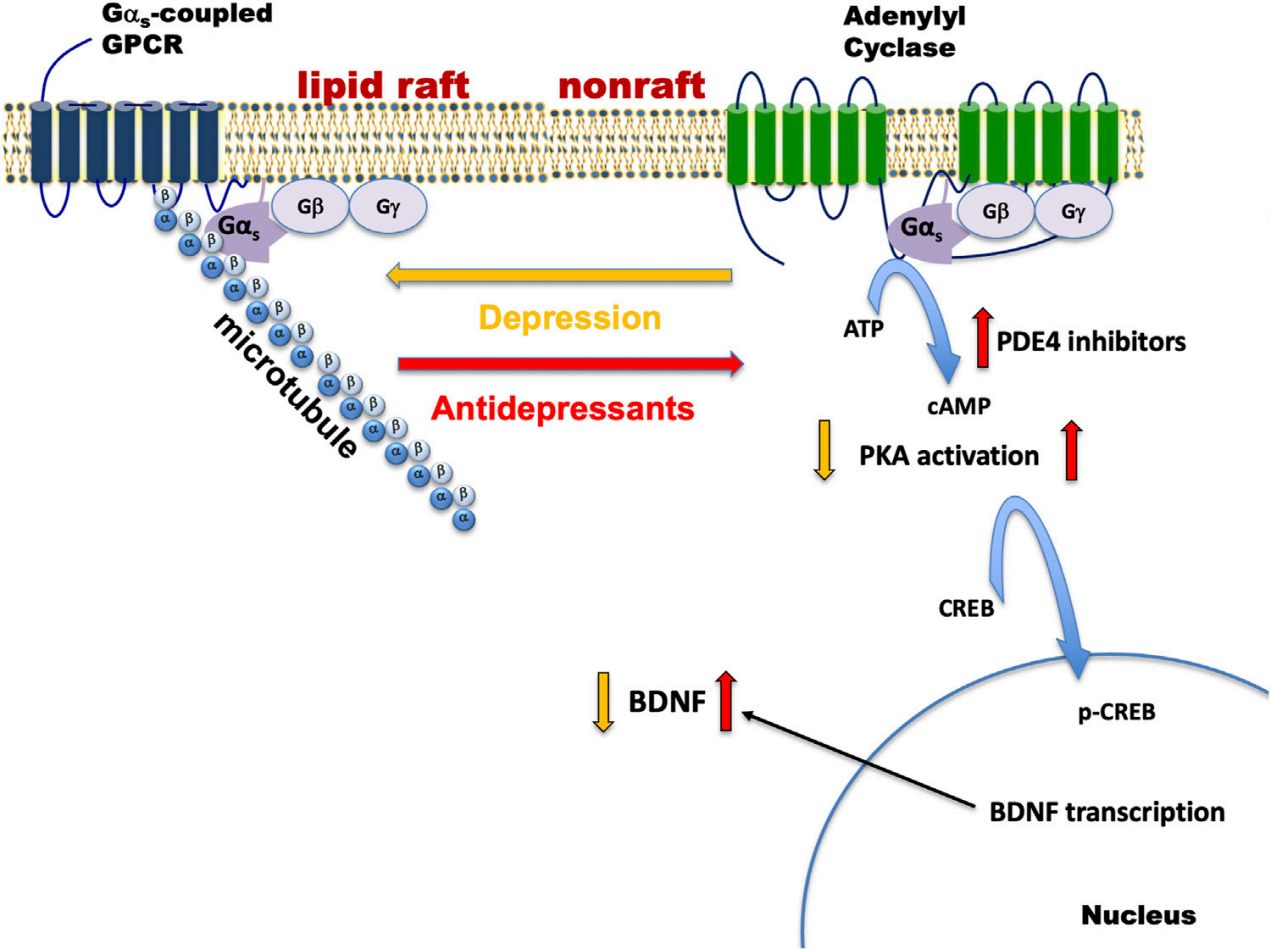
Depression and antidepressant effects on Gα_s_ plasma membrane localization. Gα_s_ is normally distributed between non-raft regions of the membrane where it moves freely and promotes neurotransmitter-activated adenylyl cyclase activity and a specialized region of the membrane rich in cholesterol (lipid raft), where the movement/adenylyl cyclase activation of Gα_s_ is impaired. During depression, Gα_s_ is enriched in the lipid raft region and it is anchored there by the structural protein, tubulin (α/β). Antidepressant treatment changes Gα_s_ such that it exits from the raft and moves to the non-raft region where it completes the process of neurotransmitter action by activating the enzyme, adenylyl cyclase. Increased Gα_s_-mediated adenylyl cyclase activity results in increased generation of cAMP, PKA activation, phosphorylation of CREB, and increases in BDNF transcription and translation. cAMP phosphodiesterase (e.g., PDE4) is another point of regulation for cAMP and inhibition here will also result in increased cAMP activity. Gα_s_-coupled GPCRs that may be relevant to depression include receptors for corticotropin releasing factor (CRF 1&2), serotonin (5-HT4,6,7), dopamine (D1), and β-adrenergic receptors. Antidepressant treatment also causes some Gα_s_ to be released from the plasma membrane, where it associates with microtubules and modifies their dynamic behavior.

We have tested a variety of cell lines including C6 glioma, PC12 pheochromocytoma, SK-N-SH neuroblastoma, as well as patient stem cell-derived neural and glial cell lines, for G protein-based antidepressant response. All compounds with antidepressant activity elicited translocation of Gα_s_ out of lipid rafts, enhanced Gα_s_–mediated cAMP generation, and slowed FRAP (fluorescence recovery after photobleaching) of GFP-Gα_s_. Collectively, antidepressant actions on Gα_s_ and adenylyl cyclase are summarized in Figure 1.

Antidepressants from all functional and chemical classes tested, promoted movement of Gα_s_ out of lipid rafts, and enhanced stimulation of adenylyl cyclase promoting increased cAMP production. Total cellular Gα_s_ content is unchanged; the shift out of lipid rafts represents a redistribution of Gα_s_. Furthermore, G proteins Gα_i_, Gα_o_, and Gα_q_ are unaffected. The redistribution of Gα_s_ out of lipid rafts occurs in a dose- and time-dependent fashion, with maximal effect occurring after approximately 3 days of antidepressant treatment, perhaps mirroring the delayed onset of effects in human subjects (which typically requires several weeks of treatment). Notably, the “rapid acting antidepressant”, ketamine, also produces these effects, and on an accelerated timeline), matching the rapid effects seen in humans.

The above findings have consistently been seen in cells of neural/glial character, either cell lines (such as C6 glioma, PC12 pheochromocytoma, and SK-N-SH neuroblastoma, or induced neural stem cells (Yu et al., 2021), while the effect has not been seen in cell lines of non-neural origin such as HEK293 and COS7. This has also raised the question of why cell lines, which cannot be considered to be “depressed” (though the tissue from which they were generated may be from a depressed subject, as in our patient stem cell-derived lines), nonetheless show an “antidepressant response.” This is a consistent empirical finding in the antidepressant responsive cell lines. Likewise, “normal” rodents respond to antidepressant administration in many behavioral tests. Selected studies reflecting the above findings are presented below.

Chen and Rasenick examined in C6 glioma the effect of chronic tricyclic antidepressant desipramine treatment for 1–5 days at 5 and 10 μM (note: 50 mM was also tested but was deleterious to cells) on membrane cAMP production in response to stimulation with nonhydrolyzable GTP analog Gpp (NH)p (direct G protein activator) or isoproterenol (Chen and Rasenick, 1995b). Membrane cAMP production was significantly enhanced in a dose- and time-dependent fashion. This effect was not seen in membranes treated acutely (at the time of assay) with desipramine. The effect of desipramine treatment on total membrane content (lipid rafts were not considered at this point) of Gα_s_, Gα_i_, Gα_o_, and Gβ was assessed by western blot, and no difference was found, compared to control. A similar contemporaneous study in rats, also by Chen and Rasenick, showed findings consistent with those in C6 glioma (Chen and Rasenick, 1995a). Here, rats treated with tricyclic antidepressants amitriptyline and desipramine were assessed for cortical membrane cAMP production and G protein disposition, as well as Gα_s_/adenylyl cyclase interaction *via* co-immunoprecipitation. Also tested were amphetamine (which elevates synaptic norepinephrine and serotonin (Berman et al., 2009), the canonical mechanism of action of most extant antidepressants, but without recognized clinical antidepressant activity) and ECT (electroconvulsive therapy). As in C6 glioma, treatment of rats with antidepressant drugs resulted in enhanced membrane cAMP production in response to Gpp (NH)p or forskolin stimulation, compared to controls. Unlike C6 glioma, no effect was seen with 1-day treatment; 21-day treatment was required, which is more similar to the extended treatment period required for clinical effects in humans. ECT (11 sessions) produced similar effects, and no effect was seen with amphetamine. These findings were consistent with an earlier study in rats, which also found increased cAMP in cortical and hippocampal membranes post chronic, but not acute, antidepressant or ECT treatment (Menkes et al., 1983). Additionally, antidepressant treatment and ECT resulted in increased adenylyl cyclase activity immunoprecipitated with Gα_s_ as measured by assay of the immunoprecipitated complex. Again consistent with the results in C6 glioma, total membrane content of Gα_s_, Gα_i_, Gα_o_ (as well as adenylyl cyclase 1and2) were unchanged by antidepressant or ECT treatments.

While the above studies revealed enhancement of cAMP production by antidepressant treatment (and ECT), without change in total G protein content, further studies noted an enhancement by antidepressant treatment upon detergent extractability of Gα_s_, bringing lipid rafts into this scheme. Toki et al. (Toki et al., 1999) treated C6 glioma for 3 days with antidepressants iprindole, amitriptyline, and fluoxetine, as well as chlorpromazine (antipsychotic drug, structurally similar to tricyclics and without clinical antidepressant activity). Membrane proteins were sequentially extracted with Triton X-100 and Triton X-114, with Triton X-100 acting upon less hydrophobic membrane regions, and Triton X-114 acting upon more hydrophobic regions (Regula et al., 1986) (now considered as nonraft and lipid raft domains) and allowing differential separation of proteins from these membrane domains. While drug treatments again did not affect total Gα_s_ content, Gα_s_ extraction by Triton X-100 was increased, and extraction by Triton X-114 decreased, by all antidepressant treatments, suggesting a change in the membrane environment of Gα_s_ subsequent to antidepressant treatment–in contemporary parlance, a redistribution of Gα_s_ from lipid raft to nonraft membrane fractions. In contrast, Gα_i_ was unaffected and treatment with non-antidepressant chlorpromazine had no effect on Gα_s_. Cortical membranes from antidepressant-treated rats also showed similar redistribution of Gα_s_. C6 glioma membranes were additionally fractionated *via* sucrose density gradient, and consecutive fractions assayed for forskolin-stimulated cAMP production. Membrane fractions from antidepressant-treated cells showing increased Gα_s_ content had significant increases in cAMP production, compared to controls. Collectively, the above studies illustrate a cellular response to chronic antidepressant treatment specifically targeting Gα_s_, but not other G proteins, with redistribution of Gα_s_ out of lipid rafts into nonraft membrane fractions, and enhanced association with adenylyl cyclase/increased cAMP production.

Creation of a fluorescent GFP-tagged Gα_s_ construct with normal membrane expression and adenylyl cyclase activation (Yu and Rasenick, 2002) and stable C6 GFP-Gα_s_ cell line allowed development of a higher-throughput assay utilizing FRAP (Czysz et al., 2015). Treatment with antidepressants and resulting redistribution of Gα_s_ out of lipid rafts results in slower lateral membrane mobility of Gα_s_, seen as a slower recovery of GFP-Gα_s_ fluorescence after photobleaching, presumably due to increased interaction between GFP-Gα_s_ and the larger, more slowly moving adenylyl cyclase molecule. Again, treatment with numerous antidepressants from varied classes resulted in significantly slowed GFP-Gα_s_ mobility (i.e., longer half-time of recovery), while psychiatric drugs lacking antidepressant activity did not alter GFP-Gα_s_ mobility. Among notable findings were the contrasting results of currently popular antidepressant escitalopram (*S*-citalopram) and its clinically inactive stereoisomer *R*-citalopram. While escitalopram showed the antidepressant-characteristic slowing of GFP-Gα_s_ mobility, *R*-citalopram had no effect, which is consistent with traditional biochemical assays of the two drugs (Zhang and Rasenick, 2010). Also consistent with past findings were the dose-and time-dependence of antidepressant-induced Gα_s_ redistribution, as well as restriction of this effect to Gα_s_ and not other G protein types.

### PDE4 inhibitors

Phosphodiesterases (PDEs) regulate the activity of cyclic nucleotides cAMP and cGMP by catalyzing their degradation into adenosine- and guanosine-5′ monophosphate, respectively. They comprise 11 isoforms, with a growing catalog of subtypes with varied subcellular localization (Delhaye and Bardoni, 2021). They display varying selectivity for cAMP and cGMP, with some targeting both (PDE1,2,3,10,11), some cGMP-selective (PDE5,6,9), and others cAMP-selective (PDE4,7,8) (Azevedo et al., 2014). Inhibitors of cAMP-targeted phosphodiesterases, particularly PDE4, have been investigated as potential antidepressants

Rolipram (ZK 62711) was identified in the 1970s as an inhibitor of phosphodiesterase (and later, as a PDE4-specific inhibitor), elevating cAMP, but not cGMP, concentrations in rat brain tissue homogenates and slices (Schwabe et al., 1976). Early studies of rolipram in rodents as a potential antidepressant examined the drug’s (and other cAMP-selective PDE inhibitors) ability to reverse reserpine-induced hypothermia and hypokinesia (reserpine inhibits vesicular monoamine transporters, depleting synaptic monoamines) and potentiate yohimbine-induced toxicity (Wachtel, 1983). Other rodent studies identified rolipram’s ability to suppress avoidance of foot shock in a rat model of depression (“Flinders sensitive line”) (Overstreet et al., 1989). Zhang et al. examined the behavioral phenotype of heterozygous and homozygous PDE4D-subtype mouse knockouts (Zhang et al., 2002). Both knockouts showed significantly reduced immobility in tail suspension- and forced swim tests, an antidepressant-like effect, with the strongest effect seen in the heterozygous knockouts. The antidepressant effect of rolipram in the forced swim test was significantly blunted in the knockout mice, suggesting subtype PDE4D may be responsible for these effects. Additional PDE4 inhibitors Ro 20–1724, ICI 63,197, and CP 67,593 have also shown antidepressant-like effects in rats (O’Donnell, 1993). Interestingly, this study also examined the effect of forskolin, which broadly activates adenylyl cyclase isoforms, increasing cellular cAMP (Insel and Ostrom, 2003; Pinto et al., 2008), finding it to lack antidepressant activity. This could reflect a requirement for a microdomain-specific enhancement of cAMP, rather than an overall increase in cellular cAMP.

Numerous clinical studies evaluated the antidepressant activity of rolipram in humans, finding it efficacious (Zeller et al., 1984), comparable to tricyclic imipramine (Bertolino et al., 1988), but less effective than tricyclic amitriptyline (Scott et al., 1991). However, clinical use of rolipram has been limited by side effects, particularly nausea and sedation (O’Donnell and Zhang, 2004).

### Other antidepressants

More recently, HDAC6 (histone deacetylase) inhibitors have been suggested as a possible new antidepressant class (Jochems et al., 2013). Unlike other HDACs, which are targeted at nuclear histones, HDAC6 acts outside of the nucleus, particularly at Lys40 of *α*-tubulin in microtubules and inhibiting deacetylation of this residue (Hubbert et al., 2002). Singh et al. compared the effects of HDAC6 inhibitor tubastatin A to traditional antidepressants SSRI escitalopram and tricyclic imipramine in C6 glioma (Singh et al., 2018). While all drugs promoted the typical redistribution of Gα_s_ out of lipid rafts and increased expression of downstream cAMP effectors phospho-CREB and BDNF, only tubastatin A promoted tubulin acetylation. So while the three compounds ultimately converge upon Gα_s_ and Gα_s_ signaling, tubulin acetylation is not a shared mechanism of action. It is also notable that Lys40 is located in the microtubule interior on *α*-tubulin (Janke and Montagnac, 2017), while Gα_s_ interacts with β-tubulin (Layden et al., 2008), suggesting acetylation does not directly interfere with Gα_s_ binding. Also notable in this regard is the recent finding that postmortem brain from depressed subjects shows decreased acetylation of membrane-associated tubulin (typically associated with lipid rafts) (Singh et al., 2020), compared to controls, as well as increased Gα_s_ localized to lipid rafts (Donati et al., 2008)).

Nasal esketamine (*S*-ketamine) has recently been approved for depression and, in contrast to traditional antidepressants which take weeks to manifest clinical effects, esketamine (as well as racemic ketamine given intravenously or sub-lingually) has almost immediate effects lasting up to a week (Yavi et al., 2022). While ketamine’s canonical target/mechanism is antagonism of the NMDA glutamate receptor, racemic ketamine metabolite (2*R*,6*R*)-hydroxynorketamine has been suggested to possess antidepressant activity in animal models, and that its antidepressant activity, as well as those of ketamine, are independent of NMDAR activity (Zanos et al., 2016). Wray et al. examined the effect of ketamine upon Gα_s_ membrane disposition and signaling of ketamine and (2*R*,6*R*)-hydroxynorketamine in C6 glioma or primary astrocytes, as well as the effect of other NMDA antagonists (Wray et al., 2018). A single ketamine treatment produced rapid (within 15 min) translocation of Gα_s_ out of lipid rafts, persisting at least 12 h and returning to baseline within 24 h, as well as enhanced cAMP production compared to control. FRAP assay also showed the characteristic slowing of GFP-Gα_s_ membrane mobility. In contrast, NMDA antagonists memantine, AP5, and MK-801 had no effect on Gα_s_ membrane localization or mobility, suggesting NMDA antagonism is insufficient to explain ketamine’s effects in this system. Likewise, knockdown of NMDA subunit GluN1 (i.e., NR1) did not inhibit ketamine’s potentiation of cAMP production. The ketamine metabolite, (2*R*, 6*R*)-hydroxynorketamine also potentiated cAMP production and slowed GFP-Gα_s_ mobility. Together, these results suggest that ketamine shares traditional antidepressants’ effects upon Gα_s_, though in a highly accelerated fashion, mirroring ketamine’s clinical timeline, and that these effects are NMDA receptor-independent (Wray et al., 2018).

## Studies with human tissue

The human data in this area are derived from studies on peripheral tissue, such as various blood cells, from living subjects, studies on postmortem brain tissue from deceased subjects, as well as noninvasive imaging studies on living subjects.

### Postmortem studies

In a series of papers, Trevor Young’s group examined changes in cAMP, CREB, and BDNF in postmortem samples from depressed patients. Dowlatshahi et al. found significantly decreased CREB immunoreactivity in temporal cortex samples from nonmedicated depressed subjects, compared to control, while depressed subjects receiving antidepressant treatment were significantly higher than the nonmedicated and did not differ from healthy control subjects (Dowlatshahi et al., 1998). Chen et al. examined postmortem hippocampus from depressed, bipolar, and schizophrenic subjects, comparing BDNF expression in unmedicated and medicated (antidepressant) subjects (Chen et al., 2001). Overall, subjects receiving antidepressants displayed significantly increased BDNF immunoreactivity across several hippocampal regions, compared to subjects not receiving antidepressants. When depressed subjects were considered separately, these hippocampal regions showed increased BDNF expression in the medicated, but did not reach significance. A third related study examined temporal/occipital cortical brain tissue from postmortem mood disorder subjects for G protein content, cAMP production, and CREB expression (Dowlatshahi et al., 1999). While G protein (Gα_s_ and Gα_i_) content did not differ among controls and depressed and bipolar subjects, forskolin-stimulated cAMP production was decreased in the depressed and bipolar samples, though not reaching significance. CREB expression did not differ among the three groups overall; however suicide subjects did have significant reductions in CREB expression compared to nonsuicide subjects.

Donati et al. examined prefrontal cortex and cerebellum of postmortem suicide subjects and found differences in detergent extractability (Triton X-100/Triton X-114) of Gα_s_, suggesting altered membrane localization of Gα_s_ in depressed suicide subjects (Donati et al., 2008). Sequential detergent extractions of cellular membranes from both brain regions showed an increased fraction of Gα_s_ localized to lipid raft vs. nonraft membrane, where it is less able to activate adenylyl cyclase (Chen and Rasenick, 1995a), consistent with the above findings in Dowlatshahi (Dowlatshahi et al., 1999).

### Blood studies

Alterations in cAMP signaling are perhaps the oldest specific biomarker of depression and response to antidepressant treatment, and these changes are seen in varied cell types. Studies reporting reduced cAMP production in samples from depressed patients extend back to the 1970s. Alterations in platelets in depression have also noted for many years, and due to their short lifespan may serve as a better model, particularly for antidepressant response, than other circulating cells. While red blood cells persist for approximately 120 days (Thiagarajan et al., 2021), and lymphocytes for at least several months (Westera et al., 2013; Jones et al., 2015), platelet lifespan is approximately 7–10 days (Josefsson et al., 2020). Because of this, platelets may be better positioned to reflect any early changes in cellular behavior as a consequence of antidepressant treatment.

An early study on platelets from depressed patients found no significant difference in platelet cAMP production in response to PGE1 and norepinephrine stimulation, compared to controls (Wang et al., 1974). However, subsequent studies have shown decreased cAMP production in platelets from depressed subjects, as well as increased cAMP production in subjects successfully treated with antidepressants. Hines and Tabakoff examined unstimulated and agonist-stimulated (CsF, forskolin, GppNHp) cAMP production in platelets from depressed subjects, finding significantly decreased cAMP production in platelets from depressed subjects (Hines and Tabakoff, 2005). Furthermore, significance increased as subjects with history of various recent drug use (drugs of abuse, analgesics, antidepressants) were excluded from the analysis due to these drugs’ effects on adenylyl cyclase activity. Overall, subjects with the lowest platelet cAMP production had a 2–6X risk of depression compared to subjects with the greatest cAMP production. Likewise, Mooney et al. examined cAMP production in mononuclear leukocytes and platelets from depressed subjects (Mooney et al., 2013). Their results revealed two subgroups of depressed patients whose demographics and medical histories were not substantially different. One depressed group (“DP-1”) showed significant differences in cAMP production compared to controls as well as the other depressed subgroup (“DP-2”), which did not significantly differ from each other: in mononuclear leukocytes, cAMP production in response to fluoride (AlF) stimulation was significantly lower, and the ratio of GTPγS/AlF-stimulated cAMP was significantly higher; for platelets, fluoride, PGE2, and PGD2 stimulation produced significantly lower cAMP, while PGE2/AlF ratio was significantly higher. Gα_i_ activity was assessed in the three groups and did not significantly differ.

More recently, Targum et al. (2022) have examined platelet PGE1-stimulated cAMP from depressed subjects before and after antidepressant treatment, with subjects stratified into “responder” or “nonresponder” groups based on HAMD_17_ (Hamilton Depression Rating Scale) rating post-treatment (Targum et al., 2022). Consistent with Hines and Tabakoff, and Mooney et al., depressed subjects had lower PGE1-stimulated cAMP production compared to normal controls at the start of treatment. Post-treatment, responders and nonresponders differed significantly, with responders showing increased PGE1-stimulated cAMP relative to basal, compared to pre-treatment values, while this value actually decreased in nonresponders. This is notable because it suggests that restoration of cAMP production is a function of clinical improvement, rather than antidepressant exposure *per se*, and as such is promising as a marker of successful antidepressant response. Because of the short platelet lifetime/high turnover, these changes may be evidenced in advance of clinical improvement and serve to guide antidepressant selection and drug changes early in treatment.

### Imaging

An interesting series of experiments approximating direct visualization of altered brain cAMP content in depressed patients, and patients treated for depression, was done by the groups of Zarate and Innis. These experiments utilized PET detection of ^11^C-labeled PDE4 inhibitor rolipram as a proxy for cAMP concentration. Briefly, PDE4 isoenzyme PDE4D3 activity is regulated by cAMP as a target of PKA phosphorylation on two serine residues, Ser^54^ and Ser^13^, with Ser^54^ phosphorylation upregulating phosphodiesterase activity. PDE4D3 activated *via* serine phosphorylation shows both increased sensitivity to rolipram inhibition (MacKenzie et al., 2002) as well as increased affinity for rolipram binding (Hoffmann et al., 1998). Expression of PDE4 isoenzymes is also enhanced by increased cellular cAMP levels (Ye et al., 2002) (Campos-Toimil et al., 2008). Thus, both expression and activity of PDE4 are regulated by cAMP. The PET-detectable binding of ^11^C-labeled rolipram provided the basis for the following studies. Fujita et al. (Fujita et al., 2012) showed consistent decreases in ^11^C-rolipram binding across 10 brain regions in unmedicated depressed patients compared to healthy controls, suggesting corresponding decreases in cAMP in these regions, reflected as decreased PDE4 expression or activity. This study was followed by Fujita et al. (Fujita et al., 2016), which examined the effect of antidepressant treatment on ^11^C-labeled rolipram binding. Again, depressed patients demonstrated decreased labeled rolipram binding in these brain regions compared to controls. After the initial PET scan, the depressed patients were treated for 2 months with SSRI antidepressants citalopram, escitalopram, or sertraline and subjected to a second PET scan. Sertraline treatment produced significant and consistent increases in ^11^C-labeled rolipram binding in the depressed patients, but the increases in rolipram binding did not correlate with improvement of depressive symptoms. These studies support the theory of cAMP changes in depression and with antidepressant treatment, and provide a technique whereby brain cAMP can be assessed *in vivo*, but also reflect the uncertainty of cAMP’s specific role in depression pathology and treatment.

Note also advances in cellular imaging related to GPCR signaling and adenylyl cyclase. Irannejad et al. (Irannejad et al., 2013) have used conformation-sensitive nanobodies to identify intracellular activation of Gα_s_. Senese and Rasenick (Senese and Rasenick, 2021) have used fluorescent cAMP reporters targeted to different cellular and membrane domains to illustrate sustained action of certain antidepressants.

## Adenylyl cyclase isoforms and neuropsychiatric disease

Several studies exist for genetic association in humans and genetic manipulation in mice. Few human neurological or psychiatric diseases are associated with mutations in adenylyl cyclase, with only AC5 specifically implicated. Sporadic mutations in *ADCY5* result in dyskinesia (Chen D.-H. et al., 2015; Ferrini et al., 2021). Several mutations have been identified; the number of established cases is very small (<500). There is no mechanism linking *ADCY5* mutations to the exaggerated muscle movements seen in this disorder. Likewise, AC5 knockout mice display Parkinsonian-type movement disorders (Iwamoto et al., 2003). In contrast, mouse models with AC5 disruption through knockout or small molecule inhibitors show protective cardiovascular or metabolic effects (Okumura et al., 2003, 2007; Zhang et al., 2018) and tend to be “health-promoting,” with increased lifespan and reduced age-related degeneration (Yan et al., 2007) The beneficial cardioprotective effects of AC5 disruption are particularly curious, given the reported predominance of AC5 (and AC6) in cardiomyocytes (Sadana and Dessauer, 2009). Studies on adenylyl cyclase isoforms and depression are summarized in Table 1.

**TABLE 1:** Animal and human studies of AC and depression.

AC isoform	Finding	References
AC1/8 double knockout (mice)	Mixed results; overall depression-like	Krishnan et al., 2008
AC3 knockout (mice)	Depression- and anxiety-like behaviors	Chen et al., 2015b; Yang et al., 2021; Liu et al., 2020
AC3 (Human)	Decreased blood AC3 transcript in depressed subjects	Redei et al., 2014
AC4 (Human)	Temporal cortex from suicide subjects shows decreased AC4 immunoreactivity andforskolin-stimulated cAMP compared to controls	Reiach et al., 1999
AC5 knockout (mice)	Mixed results; overall decreased anxiety- and depression-like behaviors	Krishnan et al., 2008
AC5 (Human)	ADCY5 related dyskinesia, not directly related to depression	Chen et al., 2015a; Ferrini et al., 2021
AC7 knockout and overexpression (mice)	Heterozygous knockdown reduced depression-like behavior (females); Overexpression increased depression-like behaviors (females); no effect in malesUpregulation of AC7 mRNA in amygdala in mouse SERT knockout depression model	Hines et al., 2006; Joeyen-Waldorf et al., 2012
AC7 (Human)	Hines et al. identified depression-associated tandem repeat and haplotype in females; Joeyen-Waldorf et al. showed upregulation of AC7 in amygdala in depressed subjects (only males tested) and identified G to T SNP associated with increased threat-related amygdala reactivity, though G allele is found in depression associated-haplotype identified in Hines et al.	Hines et al., 2006; Joeyen-Waldorf et al., 2012
AC9 (Human)	AC9 genetic polymorphisms identified, not associated with depression	Toyota et al., 2002

Studies on adenylyl cyclase in depression demonstrate no clear or consistent role of the enzyme. Though studies of depression identify decreases in cAMP in depressed subjects, both human and animal, animal knockout studies have shown depressive, antidepressive, or no effect, depending on the study as well as the specific behavioral tests employed in the study. Likewise, human studies have associated depression with both increased and decreased expression of adenylyl cyclase, with genetic polymorphisms showing varied effect as well.

### Depression and adenylyl cyclases regulated by Ca^2+^

Numerous studies have examined the role of Ca^2+^-regulated AC isoforms in the context of depression and other mood disorders, both singly and in combination. Rodent models allow genetic experimental manipulation, which is not possible in human subjects. Therefore, the most “direct” studies of AC isoform effect upon behavior have been done in rodents, typically through knockdown studies, though overexpression studies have also been done (as with AC7, *vide infra*).

Krishnan et al. contrasted the behavioral effects of AC1/8 double knockouts to that of AC5 knockouts (Krishnan et al., 2008). First, the two knockout models displayed differences in locomotor habituation, which developed later in the testing period (>60 min), as well as sex-based differences. While female mice did not show a statistical difference compared to wild type in either knockout model, male AC5 knockouts mice displayed significantly increased locomotion, while male AC1/8 double knockout mice displayed significantly decreased locomotion. Further behavioral tests including elevated plus maze, dark-light, and forced swim tests. Elevated plus maze and dark-light tests showed clear differences between the two knockout models: for both males and females, the AC5 knockout model showed a less anxiety-like phenotype, while the AC1/8 double knockout mice did not differ from wild type. In the forced swim test, AC5 knockout females showed significantly less immobility (i.e., a less “despondent” phenotype), while AC1/8 double knockout males showed a similar effect. Sucrose preference tests showed no significant effect of AC5 knockout, with significantly decreased sucrose preference (“anhedonia”) for AC1/8 double knockout males and females. Finally, social interaction tests showed decreased sociability among the AC5 knockout (males only), while both males and females showed increased sociability in the context of the AC1/8 double knockout. Overall, AC5 knockout mice displayed a less anxiety- or anti-depressive-like behavioral phenotype, while AC1/8 knockout mice displayed a more anxiety- or depressive-like behavioral phenotype, with some curious exceptions as noted above. The authors suggest that the knockouts may have divergent effects on different behaviors, such as sociability. Biochemical analysis of several brain regions was notable for decreased BDNF and TrkB (BDNF receptor) in the amygdala of AC5, but not AC1/8 knockouts, while AC1/8 knockouts showed increased BDNF signaling in the nucleus accumbens, which is also previously reported by the authors in the context of mice in a social defeat paradigm (Krishnan et al., 2007). Others (Govindarajan et al., 2006) have reported divergent effects in a transgenic mouse model overexpressing BDNF in the amygdala and hippocampus. There, mice showed increased anxiety-like behavior in open field and elevated plus tests, while showing an antidepressant-like effect in the forced swim test. Although brain cAMP was not measured in the Krishnan et al. study, simply knocking out adenylyl cyclase isoforms clearly does not lead to depression, which is characterized by decreased cAMP in the brain globally (Fujita et al., 2012, 2016), as illustrated in the divergent behavioral effects seen in the two knockout models, as well as the apparent behavioral inconsistencies noted above. Krishnan et al. (Krishnan et al., 2008) is the first of numerous studies presented herein which illustrate this “contradiction,” perhaps better viewed as the complexity of associating specific genetic variations with specific behavioral phenotypes.

### AC3

Numerous studies in rodents as well as humans have implicated AC3 in depression. Chen et al. evaluated in global, forebrain, and forebrain-targeted inducible AC3 knockouts, depressive behavioral phenotypes (Chen X. et al., 2015). Constitutive AC3 knockouts demonstrated a collection of depressive behaviors in behavioral assessments including forced swim, tail suspension, novelty-suppressed feeding/drinking, grooming, 3-chamber sociability, and nest quality. Sleep patterns were disrupted. Total brain and hippocampal volume were reduced, a finding also associated with human depression (Sapolsky, 2001). Reductions in hippocampal neural activity and long-term potentiation were also noted, as were deficits in spatial navigation/learning. Forebrain AC3 knockouts displayed a similar depressive-like phenotype in behavioral tests (though tests of anxiety such as elevated plus maze and 3-chamber sociability did not differ from wild-type) and also shared deficits memory and navigation. To rule out developmental deficits due to AC3 ablation, an inducible AC3 forebrain knockout was also considered. Again, a depression-like behavioral phenotype was observed. Together, their findings implicate AC3 as a factor in depression.

Yang and others ablated, in a mouse model, AC3 in somatostatin- and parvalbumin-expressing interneurons (Yang et al., 2021). Here, deletion of AC3 resulted in a variety of depressive- or anxious-like behaviors, but only through deletion in somatostatin-expressing interneurons. The authors note other studies implicating somatostatin interneurons in depression, including Douillard-Guilloux et al., who showed significantly diminished number of somatostatin interneurons in postmortem amygdala of depressed female subjects (Douillard-Guilloux et al., 2017). The connection to AC3, however, is unclear.

AC3 is highly expressed in olfactory cilia, and loss of AC3 results in anosmia (Wong et al., 2000) (Wang et al., 2006). Depressive (but also anxiolytic) behaviors have been reported (Ahn et al., 2016, 2018) in mouse models of anosmia chemically induced by application of zinc sulfate solution (McBride et al., 2003). Liu et al. examined behavior in mouse AC3 knockouts, olfactory epithelium-targeted AC3 knockouts, and zinc sulfate-induced anosmia mice (Liu et al., 2020). Consistent with other studies of AC3 knockouts, mice displayed increased depressive- and anxious-like behaviors compared to controls. Interestingly, the olfactory epithelium AC3 knockouts displayed similar depressive- and anxiety-like behaviors, while the zinc sulfate anosmic mice displayed primarily depressive behaviors. All three models also showed decreases in dopaminergic and glutaminergic pathways in the hippocampus, with reductions in mRNA or expressed protein for tyrosine hydroxylase, various dopamine receptors, and glutamate receptor subunit GluN2B. Again, it is unclear how AC3 ablation affects the above parameters, or how chemically-induced anosmia results in a similar behavioral and biochemical profile.

In human subjects, Redei et al. have examined blood RNA biomarkers of depression in a depressed population undergoing cognitive behavioral therapy (Redei et al., 2014). *ADCY3* expression was found to be significantly decreased in the depressed population compared to nondepressed subjects, and this difference normalized after the completion of the therapy period (although all patients did not achieve full remission). Targum et al. used platelet PGE1 stimulated cAMP production as a blood biomarker of depression as well as antidepressant response, finding significant decreases in depressed subjects compared to healthy controls, as well as increases in platelet cAMP, but only in subjects who achieved remission post-antidepressant treatment (Targum et al., 2022). This is relevant in the context of AC3, because AC3 has been reported as the primary adenylyl cyclase isoform expressed in platelets (Katsel et al., 2009). Still, questions remain regarding the relevance of AC3 for human depression (Rasenick, 2016).

### Adenylyl cyclases not regulated by Ca^2+^

#### AC4

Reiach and others have compared adenylyl cyclase expression (AC1, AC4, AC5/6) in postmortem brain (temporal cortex) from suicide, bipolar, and nondepressed control subjects *via* western blot (Reiach et al., 1999). While no difference was seen in bipolar vs normal subjects, the suicide group showed a significant decrease in AC4 immunoreactivity compared to control. Membranes from these tissues were also assayed for basal, forskolin, and GTPγS-stimulated cAMP production. Forskolin-stimulated cAMP relative to basal was significantly decreased in the suicide group compared to controls. This is consistent with others’ findings in depression, but the relevance of cAMP changes attributable to any given isoform to the overall clinical phenotype is unclear. Furthermore, antibodies available for adenylyl cyclase isoforms are of notoriously poor quality, rendering quantitation difficult.

#### AC7

A role for AC7 in depression, as well as sex-based differences, has been identified through human gene array analysis and studies of genetically altered mice. Hines et al. examined mice overexpressing AC7, as well as heterozygous AC7 knockout mice in forced swim and tail suspension tests (Hines et al., 2006). In forced swim tests, female mice overexpressing AC7 had a longer period of immobility compared to their wild-type counterparts, suggesting a depressive-like phenotype, while female heterozygous knockouts had a shorter period of immobility, suggesting an anti-depressive-like phenotype. In tail suspension tests, female mice overexpressing AC7 displayed longer immobility compared to wild type, with no effect of the heterozygous knockout. However, the performance of male mice in both behavioral tests was unaffected by either genetic manipulation. These results suggest overexpression of AC7 as promoting a depression-like phenotype, with reduced expression promoting the opposite effect, but only in female mice. The same study also examined polymorphisms in *ADCY7*, specifically a variable tetranucleotide (AACA) repeat, in the 3’ UTR of human populations in relation to depression. In particular, a seven-fold repeat of (AACA) was associated with depression in humans. The study considered separately, “family history of depression” (history of depression in a first degree relative) as well as “familial depression” (depressed subject, plus history of depression in a first-degree relative). The (AACA)_7_ repeat was significantly associated with family history of depression in females, but not in males, or females and males grouped together. For familial depression, (AACA)_7_ was significantly associated with depression in females as well as females plus males grouped together, and nearly significant for males alone (*p* = 0.08). Finally, the authors examined a series of SNPs in *ADCY7* and found no association of depression with any particular SNP, but identified a particular haplotype (TG7AT) associated with increased depression risk, and significant in females.

Joeyen-Waldorf et al. examined AC7 expression and polymorphisms in the context of human depression, as well as a SERT knockout mouse depression model (Joeyen-Waldorf et al., 2012). Both postmortem tissue from humans, as well as mouse SERT knockout mice, showed significant changes in expression of a large number of genes, with an increase of AC7 transcript seen in the amygdala in both mice and humans (note, only male humans were tested in this part of the study). The second part of the study assessed threat-related amygdala activity *via* fMRI in a population of human males plus females, in the context of rs1064448, a G to T SNP in the region of the female depression-associated haplotype identified above in Hines (Hines et al., 2006). Carriers of the T allele showed significant elevation of amygdala reactivity, compared to G homozygotes; no sex difference was observed. Interestingly, the authors note that while the T allele was associated with increased threat-related amygdala reactivity in this study, the G allele is represented in the female depression-associated TG7AT haplotype identified in Hines et al., and suggest that this apparent contradiction may be due to the difficulties in extrapolating clinical phenotypes from genetic polymorphisms, *versus* the more consistent associations seen between genetics and more “proximate” biological findings, such as the fMRI imaging data obtained in this study. Another possibility is that variety of depression phenotypes seen clinically may be associated with a variety of genetic polymorphisms.

Also notable for both studies is the association of increased AC7 expression with depression. While Joeyen-Waldorf et al. examined only mRNA, Hines et al. additionally examined AC7 protein expression and AC7-specific cAMP production, finding protein and cAMP highest for AC7 overexpression, lowest for heterozygous knockouts, and intermediate for wild-type mice. Given the large number of studies identifying decreased cAMP in brain and other cell types such as platelets in depression, as well as downstream targets of cAMP, it is counterintuitive that increased AC7 expression and cAMP production are associated with depression. Others such as Krishnan et al. have found similar results, with AC1/8 double knockout mice showing a depressive-like phenotype, and AC5 knockouts showing an anti-depressive-like phenotype (Krishnan et al., 2008). Perhaps this apparent contradiction again reflects the complexity of the combined effect of a variety of cyclase isoforms, or regional effects in cyclase isoform regulation. Finally, Price and Brust have reviewed AC7 and neuropsychiatric disorders and this review may be of interest to the reader (Price and Brust, 2019).

#### AC9

Toyota et al. examined polymorphisms in *ACDY9* in a depressed (unipolar and bipolar) population of males and females, compared to non-depressed controls (Toyota et al., 2002). Seven SNPs and one tandem repeat of (TTTA)_4 or 5_ in the 3’ UTR were identified, with one SNP (2316A>G; Ile772Met) constituting a missense mutation. The tandem repeat and missense mutation were selected for further study due to their potential for altered regulation or function. In both cases, the polymorphism was not associated with depression, compared to controls, and no difference was seen when unipolar and bipolar depression were considered separately or together. The functional consequences of the missense mutation were later examined in Small et al. through expression in HEK293 and the polymorphism demonstrated altered sensitivity to β_2_ adrenergic stimulation *via* isoproterenol (Small et al., 2003). While EC50 did not differ between the two variants, isoproterenol was significantly less efficacious in stimulating cAMP production from the Met variant. Significantly diminished efficacy was also observed upon stimulation with fluoride (direct activator of Gα_s_) as well as Mn^2+^ (direct activator of adenylyl cyclase).

## Conclusion

Consistently inconsistent, is the term best describing the studies described above on depression and adenylyl cyclase. This underscores the difficulty of translating alterations in the activity of a given adenylyl cyclase isoform to the development of a clinically significant phenotype.

While a great deal has been published on adenylyl cyclase and depression, it is difficult to develop a viable framework to house these myriad results. While it does appear that cAMP is lower in depression and that antidepressants increase overall cAMP, there is no evidence linking this to the etiology of depression or mechanism of antidepressant action. While the association between Gα_s_ and adenylyl cyclase seems relevant for depression and antidepressant therapy, the identity of the isoforms involved is yet to be determined. Further complications could be distribution of adenylyl cyclase isoforms in the many types of cells resident in the human brain and the possibility that those isoforms are unequally distributed amongst membrane microdomains (e.g. lipid rafts). Further complicating this is that this differential distribution may differ in mouse and human. Nonetheless, techniques developed with support from the BRAIN Initiative have permitted rapid advances in identifying circuitry in the brains of both mice and humans. Hopefully, a resolution will accompany the development of improved tools (e.g. opto- and chemogenetics and sophisticated fluorescent reporters) for investigation of adenylyl cyclase, its distribution, and its regulation.

## References

[B1] Adachi M. Barrot M. Autry A. E. Theobald D. Monteggia L. M. (2008). Selective loss of brain-derived neurotrophic factor in the dentate gyrus attenuates antidepressant efficacy. Biol. Psychiatry 63, 642–649. 10.1016/j.biopsych.2007.09.019 17981266 PMC2352150

[B2] Ahn S. Choi M. Kim H. Yang E. Mahmood U. Kang S.-I. (2018). Transient anosmia induces depressive-like and anxiolytic-like behavior and reduces amygdalar corticotropin-releasing hormone in a ZnSO4-induced mouse model. Chem. Senses 43, 213–221. 10.1093/chemse/bjy008 29438489

[B3] Ahn S. Shin H.-W. Mahmood U. Khalmuratova R. Jeon S.-Y. Jin H. R. (2016). Chronic anosmia induces depressive behavior and reduced anxiety via dysregulation of glucocorticoid receptor and corticotropin-releasing hormone in a mouse model. Rhinology 54, 80–87. 10.4193/Rhin15.209 26697778

[B4] Autry A. E. Adachi M. Nosyreva E. Na E. S. Los M. F. Cheng P. (2011). NMDA receptor blockade at rest triggers rapid behavioural antidepressant responses. Nature 475, 91–95. 10.1038/nature10130 21677641 PMC3172695

[B5] Azevedo M. F. Faucz F. R. Bimpaki E. Horvath A. Levy I. Alexandre R. B. de (2014). Clinical and molecular genetics of the phosphodiesterases (PDEs). Endocr. Rev. 35, 195–233. 10.1210/er.2013-1053 24311737 PMC3963262

[B6] Berman S. M. Kuczenski R. McCracken J. T. London E. D. (2009). Potential adverse effects of amphetamine treatment on brain and behavior: A review. Mol. Psychiatry 14, 123–142. 10.1038/mp.2008.90 18698321 PMC2670101

[B7] Bertolino A. Crippa D. Dio S. D. Fichte K. Musmeci G. Porro V. (1988). Rolipram versus lmipramine in inpatients with major, “minor” or atypical depressive disorder: A double-blind double-dummy study aimed at testing a novel therapeutic approach. Int. Clin. Psychopharmacol. 3, 245–253. 10.1097/00004850-198807000-00006 3153712

[B8] Björkholm C. Monteggia L. M. (2016). Bdnf - a key transducer of antidepressant effects. Neuropharmacology 102, 72–79. 10.1016/j.neuropharm.2015.10.034 26519901 PMC4763983

[B9] Blendy J. A. (2006). The role of CREB in depression and antidepressant treatment. Biol. Psychiatry 59, 1144–1150. 10.1016/j.biopsych.2005.11.003 16457782

[B10] Campos-Toimil M. Keravis T. Orallo F. Takeda K. Lugnier C. (2008). Short-term or long-term treatments with a phosphodiesterase-4 (PDE4) inhibitor result in opposing agonist-induced Ca(2+) responses in endothelial cells. Br. J. Pharmacol. 154, 82–92. 10.1038/bjp.2008.56 18311187 PMC2438981

[B11] Castrén E. Monteggia L. (2021). Brain-derived neurotrophic factor signaling in depression and antidepressant action. Biol. Psychiatry 90, 128–136. 10.1016/j.biopsych.2021.05.008 34053675

[B12] Chen B. Dowlatshahi D. MacQueen G. M. Wang J.-F. Young L. T. (2001). Increased hippocampal BDNF immunoreactivity in subjects treated with antidepressant medication. Biol. Psychiatry 50, 260–265. 10.1016/s0006-3223(01)01083-6 11522260

[B13] Chen D.-H. Méneret A. Friedman J. R. Korvatska O. Gad A. Bonkowski E. S. (2015a). ADCY5-related dyskinesia: Broader spectrum and genotype–phenotype correlations. Neurology 85, 2026–2035. 10.1212/wnl.0000000000002058 26537056 PMC4676753

[B14] Chen J. Rasenick M. M. (1995a). Chronic antidepressant treatment facilitates G protein activation of adenylyl cyclase without altering G protein content. J. Pharmacol. Exp. Ther. 275, 509–517. 7562593

[B15] Chen J. Rasenick M. M. (1995b). Chronic treatment of C6 glioma cells with antidepressant drugs increases functional coupling between a G protein (gs) and adenylyl cyclase. J. Neurochem. 64, 724–732. 10.1046/j.1471-4159.1995.64020724.x 7830066

[B16] Chen X. Luo J. Leng Y. Yang Y. Zweifel L. S. Palmiter R. D. (2015b). Ablation of type III adenylyl cyclase in mice causes reduced neuronal activity, altered sleep pattern, and depression-like phenotypes. Biol. Psychiatry 80, 836–848. 10.1016/j.biopsych.2015.12.012 26868444 PMC5972377

[B17] Conti A. C. Cryan J. F. Dalvi A. Lucki I. Blendy J. A. (2002). cAMP response element-binding protein is essential for the upregulation of brain-derived neurotrophic factor transcription, but not the behavioral or endocrine responses to antidepressant drugs. J. Neurosci. 22, 3262. 10.1523/JNEUROSCI.22-08-03262.2002 11943827 PMC6757540

[B18] Coppell A. L. Pei Q. Zetterström T. S. C. (2003). Bi-phasic change in BDNF gene expression following antidepressant drug treatment. Neuropharmacology 44, 903–910. 10.1016/s0028-3908(03)00077-7 12726822

[B19] Czysz A. H. Schappi J. M. Rasenick M. M. (2015). Lateral diffusion of Gα_s_ in the plasma membrane is decreased after chronic but not acute antidepressant treatment: Role of lipid raft and non-raft membrane microdomains. Neuropsychopharmacology 40, 766–773. 10.1038/npp.2014.256 25249058 PMC4289966

[B20] Delhaye S. Bardoni B. (2021). Role of phosphodiesterases in the pathophysiology of neurodevelopmental disorders. Mol. Psychiatry 26, 4570–4582. 10.1038/s41380-020-00997-9 33414502 PMC8589663

[B21] Donati R. J. Dwivedi Y. Roberts R. C. Conley R. R. Pandey G. N. Rasenick M. M. (2008). Postmortem brain tissue of depressed suicides reveals increased gs localization in lipid raft domains where it is less likely to activate adenylyl cyclase. J. Neurosci. 28, 3042–3050. 10.1523/jneurosci.5713-07.2008 18354007 PMC6670711

[B22] Donati R. J. Rasenick M. M. (2003). G protein signaling and the molecular basis of antidepressant action. Life Sci. 73, 1–17. 10.1016/s0024-3205(03)00249-2 12726882

[B23] Douillard‐Guilloux G. Lewis D. Seney M. L. Sibille E. (2017). Decrease in somatostatin‐positive cell density in the amygdala of females with major depression. Depress. Anxiety 34, 68–78. 10.1002/da.22549 27557481 PMC5222785

[B24] Dowlatshahi D. MacQueen G. M. Wang J. F. Young L. T. (1998). Increased temporal cortex CREB concentrations and antidepressant treatment in major depression. Lancet 352, 1754–1755. 10.1016/s0140-6736(05)79827-5 9848357

[B25] Dowlatshahi D. MacQueen G. M. Wang J. Reiach J. S. Young L. T. (1999). G protein-coupled cyclic AMP signaling in postmortem brain of subjects with mood disorders: Effects of diagnosis, suicide, and treatment at the time of death. J. Neurochem. 73, 1121–1126. 10.1046/j.1471-4159.1999.0731121.x 10461903

[B26] Duman R. S. Malberg J. Thome J. (1999). Neural plasticity to stress and antidepressant treatment. Biol. Psychiatry 46, 1181–1191. 10.1016/s0006-3223(99)00177-8 10560024

[B27] Dunham J. S. Deakin J. F. W. Miyajima F. Payton A. Toro C. T. (2009). Expression of hippocampal brain-derived neurotrophic factor and its receptors in Stanley consortium brains. J. Psychiatr. Res. 43, 1175–1184. 10.1016/j.jpsychires.2009.03.008 19376528

[B28] Dwivedi Y. Pandey G. N. (2008). Adenylyl cyclase-cyclicAMP signaling in mood disorders: Role of the crucial phosphorylating enzyme protein kinase A. Neuropsychiatr. Dis. Treat. 4, 161–176. 10.2147/ndt.s2380 18728821 PMC2515915

[B29] El-Haggar S. M. Eissa M. A. Mostafa T. M. El-Attar K. S. Abdallah M. S. (2018). The phosphodiesterase inhibitor pentoxifylline as a novel adjunct to antidepressants in major depressive disorder patients: A proof-of-concept, randomized, double-blind, placebo-controlled trial. Psychother. Psychosom. 87, 331–339. 10.1159/000492619 30205379

[B30] Ferrini A. Steel D. Barwick K. Kurian M. A. (2021). An update on the phenotype, genotype and neurobiology of ADCY5‐related disease. Mov. Disord. 36, 1104–1114. 10.1002/mds.28495 33934385

[B31] Foubert G. de Carney S. L. Robinson C. S. Destexhe E. J. Tomlinson R. Hicks C. A. (2004). Fluoxetine-induced change in rat brain expression of brain-derived neurotrophic factor varies depending on length of treatment. Neuroscience 128, 597–604. 10.1016/j.neuroscience.2004.06.054 15381288

[B32] Fujimaki K. Morinobu S. Duman R. S. (2000). Administration of a cAMP phosphodiesterase 4 inhibitor enhances antidepressant-induction of BDNF mRNA in rat Hippocampus. Neuropsychopharmacology 22, 42–51. 10.1016/s0893-133x(99)00084-6 10633490

[B33] Fujita M. Hines C. S. Zoghbi S. S. Mallinger A. G. Dickstein L. P. Liow J. S. (2012). Downregulation of brain phosphodiesterase type IV measured with 11C-(R)-Rolipram positron emission tomography in major depressive disorder. Biol. Psychiatry 72, 548–554. 10.1016/j.biopsych.2012.04.030 22677471 PMC3438357

[B34] Fujita M. Richards E. M. Niciu M. J. Ionescu D. F. Zoghbi S. S. Hong J. (2016). cAMP signaling in brain is decreased in unmedicated depressed patients and increased by treatment with a selective serotonin reuptake inhibitor. Mol. Psychiatry 22, 754–759. 10.1038/mp.2016.171 27725657 PMC5388600

[B35] Govindarajan A. Rao B. S. S. Nair D. Trinh M. Mawjee N. Tonegawa S. (2006). Transgenic brain-derived neurotrophic factor expression causes both anxiogenic and antidepressant effects. Proc. Natl. Acad. Sci. U. S. A. 103, 13208–13213. 10.1073/pnas.0605180103 16924103 PMC1559778

[B36] Hines L. M. Hoffman P. L. Bhave S. Saba L. Kaiser A. Snell L. (2006). A sex-specific role of type VII adenylyl cyclase in depression. J. Neurosci. 26, 12609–12619. 10.1523/jneurosci.1040-06.2006 17135423 PMC6674903

[B37] Hines L. M. Tabakoff B. (2005). Platelet adenylyl cyclase activity: A biological marker for major depression and recent drug use. Biol. Psychiatry 58, 955–962. 10.1016/j.biopsych.2005.05.040 16095566

[B38] Hoffmann R. Wilkinson I. R. McCallum J. F. Engels P. Houslay M. D. (1998). cAMP-specific phosphodiesterase HSPDE4D3 mutants which mimic activation and changes in rolipram inhibition triggered by protein kinase A phosphorylation of Ser-54: generation of a molecular model. Biochem. J. 333, 139–149. 10.1042/bj3330139 9639573 PMC1219566

[B39] Hubbert C. Guardiola A. Shao R. Kawaguchi Y. Ito A. Nixon A. (2002). HDAC6 is a microtubule-associated deacetylase. Nature 417, 455–458. 10.1038/417455a 12024216

[B40] Hwang J.-P. Tsai S.-J. Hong C.-J. Yang C.-H. Lirng J.-F. Yang Y.-M. (2006). The Val66Met polymorphism of the brain-derived neurotrophic-factor gene is associated with geriatric depression. Neurobiol. Aging 27, 1834–1837. 10.1016/j.neurobiolaging.2005.10.013 16343697

[B41] Insel P. A. Ostrom R. S. (2003). Forskolin as a tool for examining adenylyl cyclase expression, regulation, and G protein signaling. Cell. Mol. Neurobiol. 23, 305–314. 10.1023/a:1023684503883 12825829 PMC11530207

[B42] Irannejad R. Tomshine J. C. Tomshine J. R. Chevalier M. Mahoney J. P. Steyaert J. (2013). Conformational biosensors reveal GPCR signalling from endosomes. Nature 495, 534–538. 10.1038/nature12000 23515162 PMC3835555

[B43] Iwamoto T. Okumura S. Iwatsubo K. Kawabe J.-I. Ohtsu K. Sakai I. (2003). Motor dysfunction in type 5 adenylyl cyclase-null mice. J. Biol. Chem. 278, 16936–16940. 10.1074/jbc.c300075200 12665504

[B44] Janke C. Montagnac G. (2017). Causes and consequences of microtubule acetylation. Curr. Biol. 27, R1287–R1292. 10.1016/j.cub.2017.10.044 29207274

[B45] Jochems J. Boulden J. Lee B. G. Blendy J. A. Jarpe M. Mazitschek R. (2013). Antidepressant-like properties of novel HDAC6-selective inhibitors with improved brain bioavailability. Neuropsychopharmacology 39, 389–400. 10.1038/npp.2013.207 23954848 PMC3870780

[B46] Joeyen-Waldorf J. Nikolova Y. S. Edgar N. Walsh C. Kota R. Lewis D. A. (2012). Adenylate cyclase 7 is implicated in the biology of depression and modulation of affective neural circuitry. Biol. Psychiatry 71, 627–632. 10.1016/j.biopsych.2011.11.029 22264442 PMC3307939

[B47] Jones D. D. Wilmore J. R. Allman D. (2015). Cellular dynamics of memory B cell populations: IgM+ and IgG+ memory B cells persist indefinitely as quiescent cells. J. Immunol. 195, 4753–4759. 10.4049/jimmunol.1501365 26438523 PMC4637268

[B48] Josefsson E. C. Vainchenker W. James C. (2020). Regulation of platelet production and life span: Role of bcl-xL and potential implications for human platelet diseases. Int. J. Mol. Sci. 21, 7591. 10.3390/ijms21207591 33066573 PMC7589436

[B49] Katsel P. L. Tagliente T. M. Schwarz T. E. Craddock-Royal B. D. Patel N. D. Maayani S. (2009). Molecular and biochemical evidence for the presence of Type III adenylyl cyclase in human platelets. Platelets 14, 21–33. 10.1080/0953710021000062905 12623444

[B50] Kennedy S. H. Eisfeld B. S. (2007). Agomelatine and its therapeutic potential in the depressed patient. Neuropsychiatr. Dis. Treat. 3, 423–428. 19300571 PMC2655086

[B51] Krishnan V. Graham A. Mazei-Robison M. S. Lagace D. C. Kim K.-S. Birnbaum S. (2008). Calcium-sensitive adenylyl cyclases in depression and anxiety: Behavioral and biochemical consequences of isoform targeting. Biol. Psychiatry 64, 336–343. 10.1016/j.biopsych.2008.03.026 18468583 PMC2580057

[B52] Krishnan V. Han M.-H. Graham D. L. Berton O. Renthal W. Russo S. J. (2007). Molecular adaptations underlying susceptibility and resistance to social defeat in brain reward regions. Cell 131, 391–404. 10.1016/j.cell.2007.09.018 17956738

[B53] Layden B. T. Saengsawang W. Donati R. J. Yang S. Mulhearn D. C. Johnson M. E. (2008). Structural model of a complex between the heterotrimeric G protein, Gsalpha, and tubulin. Biochim. Biophys. Acta 1783, 964–973. 10.1016/j.bbamcr.2008.02.017 18373982 PMC2453506

[B54] Liu X. Zhou Y. Li S. Yang D. Jiao M. Liu X. (2020). Type 3 adenylyl cyclase in the main olfactory epithelium participates in depression-like and anxiety-like behaviours. J. Affect. Disord. 268, 28–38. 10.1016/j.jad.2020.02.041 32158004

[B55] MacKenzie S. J. Baillie G. S. McPhee I. MacKenzie C. Seamons R. McSorley T. (2002). Long PDE4 cAMP specific phosphodiesterases are activated by protein kinase A-mediated phosphorylation of a single serine residue in Upstream Conserved Region 1 (UCR1). Br. J. Pharmacol. 136, 421–433. 10.1038/sj.bjp.0704743 12023945 PMC1573369

[B56] Malberg J. E. Blendy J. A. (2005). Antidepressant action: To the nucleus and beyond. Trends Pharmacol. Sci. 26, 631–638. 10.1016/j.tips.2005.10.005 16246434

[B57] Matveychuk D. Thomas R. K. Swainson J. Khullar A. MacKay M.-A. Baker G. B. (2020). Ketamine as an antidepressant: Overview of its mechanisms of action and potential predictive biomarkers. Ther. Adv. Psychopharmacol. 10, 2045125320916657. 10.1177/2045125320916657 32440333 PMC7225830

[B58] McBride K. Slotnick B. Margolis F. L. (2003). Does intranasal application of zinc sulfate produce anosmia in the mouse? An olfactometric and anatomical study. Chem. Senses 28, 659–670. 10.1093/chemse/bjg053 14627534

[B59] Menkes D. B. Rasenick M. M. Wheeler M. A. Bitensky M. W. (1983). Guanosine triphosphate activation of brain adenylate cyclase: Enhancement by long-term antidepressant treatment. Science 219, 65–67. 10.1126/science.6849117 6849117

[B60] Moncrieff J. Cooper R. E. Stockmann T. Amendola S. Hengartner M. P. Horowitz M. A. (2022). The serotonin theory of depression: A systematic umbrella review of the evidence. Mol. Psychiatry 14, 1. 10.1038/s41380-022-01661-0 PMC10618090 35854107

[B61] Mooney J. J. Samson J. A. McHale N. L. Pappalarado K. M. Alpert J. E. Schildkraut J. J. (2013). Increased gsα within blood cell membrane lipid microdomains in some depressive disorders: An exploratory study. J. Psychiatr. Res. 47, 706–711. 10.1016/j.jpsychires.2013.02.005 23490066 PMC3669544

[B62] O’Donnell J. M. (1993). Antidepressant-like effects of rolipram and other inhibitors of cyclic adenosine monophosphate phosphodiesterase on behavior maintained by differential reinforcement of low response rate. J. Pharmacol. Exp. Ther. 264, 1168–1178. 8383740

[B63] O’Donnell J. M. Zhang H.-T. (2004). Antidepressant effects of inhibitors of cAMP phosphodiesterase (PDE4). Trends Pharmacol. Sci. 25, 158–163. 10.1016/j.tips.2004.01.003 15019272

[B64] Okumura S. Takagi G. Kawabe J. Yang G. Lee M.-C. Hong C. (2003). Disruption of type 5 adenylyl cyclase gene preserves cardiac function against pressure overload. Proc. Natl. Acad. Sci. U. S. A. 100, 9986–9990. 10.1073/pnas.1733772100 12904575 PMC187910

[B65] Okumura S. Vatner D. E. Kurotani R. Bai Y. Gao S. Yuan Z. (2007). Disruption of type 5 adenylyl cyclase enhances desensitization of cyclic adenosine monophosphate signal and increases Akt signal with chronic catecholamine stress. Circulation 116 (16), 1776–1783. 10.1161/CIRCULATIONAHA.107.698662 17893275

[B66] Overstreet D. H. Double K. Schiller G. D. (1989). Antidepressant effects of rolipram in a genetic animal model of depression: Cholinergic supersensitivity and weight gain. Pharmacol. Biochem. Behav. 34, 691–696. 10.1016/0091-3057(89)90260-8 2623026

[B67] Pinto C. Papa D. Hubner M. Mou T. C. Lushington G. H. Seifert R. (2008). Activation and inhibition of adenylyl cyclase isoforms by forskolin analogs. J. Pharmacol. Exp. Ther. 325, 27–36. 10.1124/jpet.107.131904 18184830

[B68] Price T. Brust T. F. (2019). Adenylyl cyclase 7 and neuropsychiatric disorders: A new target for depression? Pharmacol. Res. 143, 106–112. 10.1016/j.phrs.2019.03.015 30904753

[B69] Rafa–Zabłocka K. Kreiner G. Bagińska M. Kuśmierczyk J. Parlato R. Nalepa I. (2017). Transgenic mice lacking CREB and CREM in noradrenergic and serotonergic neurons respond differently to common antidepressants on tail suspension test. Sci. Rep. 7, 13515. 10.1038/s41598-017-14069-6 29044198 PMC5647346

[B70] Rasenick M. M. (2016). Depression and adenylyl cyclase - sorting out the signals. Biol. Psychiatry 80, 812–814. 10.1016/j.biopsych.2016.09.021 27968725 PMC6126898

[B71] Redei E. E. Andrus B. M. Kwasny M. J. Seok J. Cai X. Ho J. (2014). Blood transcriptomic biomarkers in adult primary care patients with major depressive disorder undergoing cognitive behavioral therapy. Transl. Psychiatry 4, e442. 10.1038/tp.2014.66 25226551 PMC4198533

[B72] Regula C. S. Sager P. R. Berlin R. D. (1986). Membrane tubulin. Ann. N. Y. Acad. Sci. 466, 832–842. 10.1111/j.1749-6632.1986.tb38466.x 3460456

[B73] Reiach J. S. Li P. P. Warsh J. J. Kish S. J. Young L. T. (1999). Reduced adenylyl cyclase immunolabeling and activity in postmortem temporal cortex of depressed suicide victims. J. Affect. Disord. 56, 141–151. 10.1016/s0165-0327(99)00048-8 10701471

[B74] Sadana R. Dessauer C. W. (2009). Physiological roles for G protein-regulated adenylyl cyclase isoforms: Insights from knockout and overexpression studies. Neurosignals. 17, 5–22. 10.1159/000166277 18948702 PMC2790773

[B75] Sapolsky R. M. (2001). Depression, antidepressants, and the shrinking hippocampus. Proc. Natl. Acad. Sci. U. S. A. 98, 12320–12322. 10.1073/pnas.231475998 11675480 PMC60045

[B76] Schwabe U. Miyake M. Ohga Y. Daly J. W. (1976). 4-(3-Cyclopentyloxy-4-methoxyphenyl)-2-pyrrolidone (ZK 62711): A potent inhibitor of adenosine cyclic 3’, 5’-monophosphate phosphodiesterases in homogenates and tissue slices from rat brain. Mol. Pharmacol. 12, 900–910. 187926

[B77] Scott A. I. F. Perini A. F. Shering P. A. Whalley L. J. (1991). In-patient major depression: Is rolipram as effective as amitriptyline? Eur. J. Clin. Pharmacol. 40, 127–129. 10.1007/bf00280065 2065693

[B78] Senese N. B. Rasenick M. M. (2021). Antidepressants produce persistent g*α* _s_-associated signaling changes in lipid rafts after drug withdrawal. Mol. Pharmacol. 100, 66–81. MOLPHARM-AR-2020-000226. 10.1124/molpharm.120.000226 34011569 PMC8382257

[B79] Singh H. Chmura J. Bhaumik R. Pandey G. N. Rasenick M. M. (2020). Membrane-associated α-tubulin is less acetylated in postmortem prefrontal cortex from depressed subjects relative to controls: Cytoskeletal dynamics, HDAC6, and depression. J. Neurosci. 40, 4033–4041. 10.1523/jneurosci.3033-19.2020 32284336 PMC7219287

[B80] Singh H. Wray N. Schappi J. M. Rasenick M. M. (2018). Disruption of lipid-raft localized gαs/tubulin complexes by antidepressants: A unique feature of HDAC6 inhibitors, SSRI and tricyclic compounds. Neuropsychopharmacology 43, 1481–1491. 10.1038/s41386-018-0016-x 29463911 PMC5983546

[B81] Small K. M. Brown K. M. Theiss C. T. Seman C. A. Weiss S. T. Liggett S. B. (2003). An Ile to Met polymorphism in the catalytic domain of adenylyl cyclase type 9 confers reduced beta2-adrenergic receptor stimulation. Pharmacogenetics 13, 535–541. 10.1097/00008571-200309000-00002 12972952

[B82] Sulser F. Gillespie D. D. Mishra R. Manier D. H. (1984). Desensitization by antidepressants of central norepinephrine receptor systems coupled to adenylate cyclase. Ann. N. Y. Acad. Sci. 430, 91–101. 10.1111/j.1749-6632.1984.tb14500.x 6331266

[B83] Targum S. D. Schappi J. Koutsouris A. Bhaumik R. Rapaport M. H. Rasgon N. (2022). A novel peripheral biomarker for depression and antidepressant response. Mol. Psychiatry 27, 1640–1646. 10.1038/s41380-021-01399-1 34969978 PMC9106819

[B84] Thiagarajan P. Parker C. J. Prchal J. T. (2021). How do red blood cells die? Front. Physiol. 12, 655393. 10.3389/fphys.2021.655393 33790808 PMC8006275

[B85] Toki S. Donati R. J. Rasenick M. M. (1999). Treatment of C6 glioma cells and rats with antidepressant drugs increases the detergent extraction of G(s alpha) from plasma membrane. J. Neurochem. 73, 1114–1120. 10.1046/j.1471-4159.1999.0731114.x 10461902

[B86] Toyota T. Hattori E. Meerabux J. Yamada K. Saito K. Shibuya H. (2002). Molecular analysis, mutation screening, and association study of adenylate cyclase type 9 gene (ADCY9) in mood disorders. Am. J. Med. Genet. 114, 84–92. 10.1002/ajmg.10117 11840511

[B87] Verhagen M. Meij A. van der Deurzen P. A. M. van Janzing J. G. E. Arias-Vásquez A. Buitelaar J. K. (2010). Meta-analysis of the BDNF Val66Met polymorphism in major depressive disorder: Effects of gender and ethnicity. Mol. Psychiatry 15, 260–271. 10.1038/mp.2008.109 18852698

[B88] Wachtel H. (1983). Potential antidepressant activity of rolipram and other selective cyclic adenosine 3′, 5′-monophosphate phosphodiesterase inhibitors. Neuropharmacology 22, 267–272. 10.1016/0028-3908(83)90239-3 6302550

[B89] Wang Y.-C. Pandey G. N. Mendels J. Frazer A. (1974). Platelet adenylate cyclase responses in depression: Implications for a receptor defect. Psychopharmacologia 36, 291–300. 10.1007/bf00422561 4367922

[B90] Wang Z. Sindreu C. B. Li V. Nudelman A. Chan G. C.-K. Storm D. R. (2006). Pheromone detection in male mice depends on signaling through the type 3 adenylyl cyclase in the main olfactory epithelium. J. Neurosci. 26, 7375–7379. 10.1523/jneurosci.1967-06.2006 16837584 PMC6674185

[B91] Westera L. Drylewicz J. Braber I. den Mugwagwa T. Maas I. van der Kwast L. (2013). Closing the gap between T-cell life span estimates from stable isotope-labeling studies in mice and humans. Blood 122, 2205–2212. 10.1182/blood-2013-03-488411 23945154

[B92] Wong S. T. Trinh K. Hacker B. Chan G. C. K. Lowe G. Gaggar A. (2000). Disruption of the type III adenylyl cyclase gene leads to peripheral and behavioral anosmia in transgenic mice. Neuron 27, 487–497. 10.1016/s0896-6273(00)00060-x 11055432

[B93] Wray N. H. Schappi J. M. Singh H. Senese N. B. Rasenick M. M. (2018). NMDAR-independent, cAMP-dependent antidepressant actions of ketamine. Mol. Psychiatry 24, 1833–1843. 10.1038/s41380-018-0083-8 29895894 PMC8011999

[B94] Yan L. Vatner D. E. O’Connor J. P. Ivessa A. Ge H. Chen W. (2007). Type 5 adenylyl cyclase disruption increases longevity and protects against stress. Cell 130, 247–258. 10.1016/j.cell.2007.05.038 17662940

[B95] Yang X.-Y. Ma Z.-L. Storm D. R. Cao H. Zhang Y.-Q. (2021). Selective ablation of type 3 adenylyl cyclase in somatostatin-positive interneurons produces anxiety- and depression-like behaviors in mice. World J. Psychiatry 11, 35–49. 10.5498/wjp.v11.i2.35 33643860 PMC7896247

[B96] Yavi M. Lee H. Henter I. D. Park L. T. Zarate C. A. (2022). Ketamine treatment for depression: A review. Discov. Ment. Health 2, 9. 10.1007/s44192-022-00012-3 35509843 PMC9010394

[B97] Ye Y. Conti M. Houslay M. D. Farooqui S. M. Chen M. O’Donnell J. M. (2002). Noradrenergic activity differentially regulates the expression of rolipram-sensitive, high-affinity cyclic AMP phosphodiesterase (PDE4) in rat brain. J. Neurochem. 69, 2397–2404. 10.1046/j.1471-4159.1997.69062397.x 9375671

[B98] Yu J.-Z. Rasenick M. M. (2002). Real-time visualization of a fluorescent G(alpha)(s): Dissociation of the activated G protein from plasma membrane. Mol. Pharmacol. 61, 352–359. 10.1124/mol.61.2.352 11809860

[B99] Yu J.-Z. Wang J. Sheridan S. D. Perlis R. H. Rasenick M. M. (2021). N-3 polyunsaturated fatty acids promote astrocyte differentiation and neurotrophin production independent of cAMP in patient-derived neural stem cells. Mol. Psychiatry 26, 4605–4615. 10.1038/s41380-020-0786-5 32504049 PMC10034857

[B100] Zanos P. Moaddel R. Morris P. J. Georgiou P. Fischell J. Elmer G. I. (2016). NMDAR inhibition-independent antidepressant actions of ketamine metabolites. Nature 533, 481–486. 10.1038/nature17998 27144355 PMC4922311

[B101] Zeller E. Stief H. Pflug B. Sastre-y-Hernández M. (1984). Results of a phase II study of the antidepressant effect of rolipram. Pharmacopsychiatry 17, 188–190. 10.1055/s-2007-1017435 6393150

[B102] Zhang H.-T. Huang Y. Jin S.-L. C. Frith S. A. Suvarna N. Conti M. (2002). Antidepressant-like profile and reduced sensitivity to rolipram in mice deficient in the PDE4D phosphodiesterase enzyme. Neuropsychopharmacology 27, 587–595. 10.1016/s0893-133x(02)00344-5 12377395

[B103] Zhang J. Levy D. Oydanich M. Bravo C. Yoon S. Vatner D. E. (2018). A novel adenylyl cyclase type 5 inhibitor that reduces myocardial infarct size even when administered after coronary artery reperfusion. J. Mol. Cell. Cardiol. 121, 13–15. 10.1016/j.yjmcc.2018.05.014 29800555 PMC6103820

[B104] Zhang L. Rasenick M. M. (2010). Chronic treatment with escitalopram but not R-citalopram translocates galpha(s) from lipid raft domains and potentiates adenylyl cyclase: A 5-hydroxytryptamine transporter-independent action of this antidepressant compound. J. Pharmacol. Exp. Ther. 332, 977–984. 10.1124/jpet.109.162644 19996298 PMC2835448

[B105] Zhao Y. Zhang H.-Ti. O’Donnell J. M. (2003). Antidepressant-induced increase in high-affinity rolipram binding sites in rat brain: Dependence on noradrenergic and serotonergic function. J. Pharmacol. Exp. Ther. 307, 246–253. 10.1124/jpet.103.053215 12954819

